# Naturally Occurring Antioxidant Therapy in Alzheimer’s Disease

**DOI:** 10.3390/antiox11020213

**Published:** 2022-01-23

**Authors:** Andrila E. Collins, Tarek M. Saleh, Bettina E. Kalisch

**Affiliations:** Department of Biomedical Sciences and Collaborative Specialization in Neuroscience Program, University of Guelph, Guelph, ON N1G 2W1, Canada; andrila@uoguelph.ca (A.E.C.); tsaleh@uoguelph.ca (T.M.S.)

**Keywords:** antioxidants, oxidative stress, amyloid-beta, Alzheimer’s disease, clinical trials

## Abstract

It is estimated that the prevalence rate of Alzheimer’s disease (AD) will double by the year 2040. Although currently available treatments help with symptom management, they do not prevent, delay the progression of, or cure the disease. Interestingly, a shared characteristic of AD and other neurodegenerative diseases and disorders is oxidative stress. Despite profound evidence supporting the role of oxidative stress in the pathogenesis and progression of AD, none of the currently available treatment options address oxidative stress. Recently, attention has been placed on the use of antioxidants to mitigate the effects of oxidative stress in the central nervous system. In preclinical studies utilizing cellular and animal models, natural antioxidants showed therapeutic promise when administered alone or in combination with other compounds. More recently, the concept of combination antioxidant therapy has been explored as a novel approach to preventing and treating neurodegenerative conditions that present with oxidative stress as a contributing factor. In this review, the relationship between oxidative stress and AD pathology and the neuroprotective role of natural antioxidants from natural sources are discussed. Additionally, the therapeutic potential of natural antioxidants as preventatives and/or treatment for AD is examined, with special attention paid to natural antioxidant combinations and conjugates that are currently being investigated in human clinical trials.

## 1. Introduction

Mild declines in cognitive and motor abilities are common aspects of human aging. However, the development of neurodegenerative diseases and neurological conditions is not. Interestingly, although the brain is arguably the most essential organ in the human body, it remains susceptible to its own degradation. As a highly metabolically active organ, the brain’s oxygen demand is high [1]. As a result, free radicals are produced as the brain’s requirement for oxygen increases [2]. The brain contains high amounts of polyunsaturated fatty acids, which are quickly oxidized by reactive oxygen species (ROS) but lacks essential enzymes that metabolize several toxic oxygen-containing reactants into harmless compounds [3]. This susceptibility to oxidative damage is observed in several neurodegenerative diseases. Alzheimer’s disease (AD), Huntington’s disease (HD), Parkinson’s disease (PD), amyotrophic lateral sclerosis (ALS) and stroke, among many others (see Table 1), possess this shared pathology of oxidative stress. Recently, considerable attention has been placed on the use of naturally occurring (non-synthetic) antioxidants to mitigate the effects of oxidative stress in the central nervous system (CNS). More recently, the concept of combination antioxidant therapy has been explored as a novel approach to preventing and treating neurodegenerative conditions that present oxidative stress as a contributing factor to the pathogenesis and/or progression of the disease. This review explores the relationship between AD pathology and oxidative stress and the therapeutic potential of natural antioxidants as preventatives and/or treatments for AD, with an emphasis on natural antioxidant combinations and conjugates that are currently being investigated in human clinical trials.

## 2. Antioxidants and Oxidative Stress

Antioxidants are compounds that protect the body from damage due to oxidative stress. Oxidative stress occurs when there is an imbalance between antioxidants and the production and accumulation of ROS [reviewed in 4]. ROS can be defined as oxygen-containing reactive molecules that are endogenously generated through mitochondrial oxygen metabolism [36]. ROS can also be produced through interplay with exogenous substances such as xenobiotic compounds [36]. This collective term includes compounds such as hydrogen peroxide (H_2_O_2_), superoxide (O_2_^•−^), hydroxyl radical (^•^OH), nitric oxide (^•^NO) and singlet oxygen (^1^O_2_) [37]. During oxidative stress, an insufficient or dysfunctional antioxidant defence system permits damage to important cellular structures such as proteins, lipids, and nucleic acids [37,38], and is implicated in several pathologies of neurodegeneration [38] and aging [39]. Although ROS are damaging in excess, ROS maintain several endogenous functions at low levels. Under normal circumstances, low levels of ROS are produced through ordinary aerobic metabolism and any damage to cells is rapidly repaired through deployment of the antioxidant defence system [40]. ROS perform a critical role in cellular signalling processes, also known as redox signalling [41]. Therefore, to maintain adequate cellular homeostasis, a balance must be established between the production and depletion of ROS. This occurs through the protective mechanisms of antioxidants, which limit the damage induced by ROS and the eventual development of diseases and accelerated aging [39].

### 2.1. Antioxidant Classification and Mechanisms of Action

Antioxidants can be classified into two main categories: natural antioxidants and synthetic antioxidants. Synthetic antioxidants are artificially generated using a variety of chemical synthesis techniques [40,41]. Natural antioxidants are found in plants and animals and perform various biological roles, including but not limited to anti-inflammatory, anticancer and antiaging effects [42,43,44,45]. Natural antioxidants can be further divided into enzymatic and non-enzymatic antioxidants.

#### 2.1.1. Enzymatic Antioxidants

**Figure 1:**
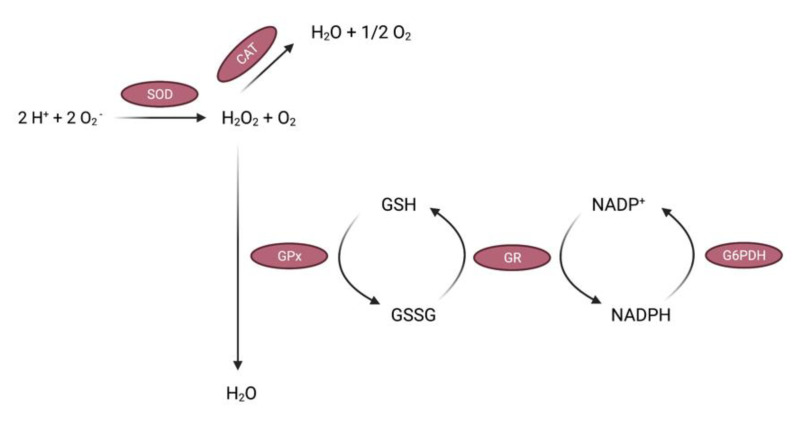
Schematic representation of enzymatic antioxidant mechanisms of action. Created with BioRender.com. SOD, superoxide dismutase; CAT, catalase; GPx, glutathione peroxidase; GR, glutathione reductase; G6PDH, glucose-6-phosphate dehydrogenase.

Enzymatic antioxidants are enzymes produced within the body that possess free radical scavenging abilities and perform antioxidant functions. This group includes primary enzymes such as superoxide dismutase (SOD), catalase (CAT), and glutathione peroxidase (GPx), and secondary enzymes including glutathione reductase (GR) and glucose-6-phosphate dehydrogenase (G6PDH) [46]. SOD eliminates O_2_^•−^ radicals by catalyzing the reduction of O_2_^•−^ anions to H_2_O_2_ [15]. SOD is present in its many forms in several cellular locations, including the cytosol (Cu-Zn SOD1 or SOD1), mitochondria (Mn SOD or SOD2) and the extracellular space (SOD3) [47]. In turn, CAT is responsible for the decomposition of H_2_O_2_ into water and oxygen molecules [48]. In the absence of CAT, H_2_O_2_ can react with metal ions to form toxic ^•^OH radicals that further perpetuate the effects of oxidative stress [49]. GPx refers to a family of selenocysteine-containing enzymes, including GPx-1, which is predominantly found in the cytoplasm, that utilize glutathione (GSH) as a co-substrate to catalyze the reduction of H_2_O_2_ to water and oxidized glutathione (GSSG), and reduce other hydroperoxide substrates to alcohols [50,51]. It was suggested that a cooperative activity between CAT and GPx is required to achieve cellular protection against harmful peroxides [52]. However, differences in the antioxidant capacity of these compounds regarding the rate of removal and the capacity to ravage H_2_O_2_ were also identified [53]. GSSG is then reduced to GSH by nicotinamide adenine dinucleotide phosphate (NADPH). G6PDH, which produces NADPH, and GR, which recycles GSSG using NADPH, are considered secondary enzymatic antioxidants (Figure 1). The processes involved in the breakdown and elimination of elevated/toxic oxidative compounds may also include the presence of essential cofactors such as zinc (Zn), copper (Cu), iron (Fe), selenium (Se) and manganese (Mn) [54].

#### 2.1.2. Non-Enzymatic Antioxidants

Non-enzymatic antioxidants can further be divided into groups of vitamins, carotenoids, polyphenols, and minerals [55]. Vitamins are a group of micronutrients that cannot be produced within the body; hence, they require supplementation through the diet [56]. Vitamins perform various functions within the body that are vital for human health and metabolism and are categorized into two groups based on solubility: fat-soluble vitamins and water-soluble vitamins [56]. Fat-soluble vitamins include vitamins A, D, E and K, which are dissolved in fat prior to their absorption into the bloodstream [56]. Water-soluble vitamins include the group of B-complex vitamins and vitamin C, which are dissolved in water [56].

##### Vitamin A

Sources of vitamin A include dietary supplements, animal products such as fish, meat, poultry, and dairy products, as well as plant products including fruits and vegetables that contain the provitamin A carotenoids (described below) such as beta-carotene, which is endogenously converted to vitamin A [57]. Vitamin A maintains essential roles in vision and synaptic function [58], bone growth and development [59], gene expression [60], cell division [61], reproduction [62], the maintenance of epithelial cells in respiratory, intestinal and urinary tracts, and a healthy immune system [63,64].

##### Vitamin D

Vitamin D, also known as vitamin D_3_ (cholecalciferol) and vitamin D_2_ (ergocalciferol), is predominantly produced endogenously within the skin from the provitamins 7-dehydrocholesterol (7-DHC) and ergosterol [65]. Skin exposure to ultraviolet-B stimulates the synthesis of pre-vitamin D_3_, followed by thermal isomerization, producing vitamin D_3_ [65]. Vitamins D_3_ and D_2_ can be obtained from the diet in supplements and fortified foods. Dietary vitamin D is predominantly absorbed in the small intestine by chylomicrons, before entering the lymphatic system and then the bloodstream [66]. Once in the bloodstream, from skin or intestinal absorption, vitamin D is converted into 25-hydroxyvitamin D in the liver, which undergoes further conversion to its active form 1,25-dihydrovitamin D in the kidneys [67,68,69]. Both compounds circulate in the blood bound to the vitamin D-binding protein. Once released from vitamin D-binding protein at tissues sites, 1,25-dihydrovitamin D binds to intracellular vitamin D receptors to elicit various metabolic functions throughout the body such as cell differentiation and proliferation, and calcium and phosphorus homeostasis [70,71].

##### Vitamin E

Vitamin E (α-tocopherol) acts as an antioxidant by protecting membrane components such as polyunsaturated fatty acids from lipid peroxidation by free radicals. Notably, vitamin E has been found to protect low-density lipoproteins from oxidation [72] and is present in high levels within the membranes of red blood cells, mitochondria, and endoplasmic reticulum [73,74,75,76]. Since vitamin E is predominantly synthesized in plants, it can be found in plant products such as nuts, seeds, vegetable oils and leafy green vegetables [77]. Vitamin E maintains various other biological functions, in addition to its role as an antioxidant, including its impact on signal transduction and gene expression [78], and its capacity to regulate enzymatic activity such as protein kinase C, which is important for regulating processes such as cell proliferation and inflammatory responses [79,80,81,82]. The effects of vitamin E may also differ between the four isoforms (α, β, γ and δ), with some studies reporting contradictory effects between these isoforms [82,83]. The ability of vitamin E to donate protons, and thereby saturate and detoxify unpaired electrons on highly reactive radicals such as ^•^OH, support its recognition as a potent antioxidant [84]. The importance of vitamin E in brain health is observed in its ability to inhibit the production and progression of chain reactions that lead to lipid peroxidation by hindering the oxidation of unsaturated side chains present within lipid membranes [85]. This role highlights vitamin E as a potential therapeutic agent for neurological conditions, particularly those characterized by oxidative damage. For example, cerebral ischemia and subsequent infarction are consequences of oxidative stress in which vitamin E provides protection [86]. In addition to scavenging free radicals, studies report that vitamin E reduces the toxic effects of ^•^NO by converting it to a less toxic nitrite ester in vitro and decreases the production of ^•^NO and O_2_^•−^ within the brain [87,88].

##### Vitamin K

Vitamin K is bio-actively found in two forms, vitamins K_1_ and K_2_. Vitamin K_1_ (phylloquinone) is predominately found in green leafy plants and can also be found in animals and further converted to vitamin K_2_ (menaquinone) by anaerobic gut bacteria in animals [89,90]. Vitamin K_2_ is primarily known for its role in blood-clotting by synthesizing coagulation proteins. It exerts its primary function by creating gamma-carboxyglutamate residues during the production of clotting factors by combining glutamate residues with carboxylic acids [91]. The addition of two carboxylic acid groups to an individual carbon present within a gamma-carboxyglutamate residue permits it to chelate calcium ions. Calcium ion binding is essential for vitamin K-dependent clotting factors, resulting in continued clotting cascades [92]. In a process called the vitamin K cycle, vitamin K is reduced to its metabolic form, vitamin K hydroquinone, by vitamin K epoxide reductase (VKOR), within the cell [93]. In turn, vitamin K hydroquinone is oxidized by vitamin K-dependent carboxylase. This enzyme then carboxylates glutamate residues to gamma-carboxyglutamate residues, ultimately producing vitamin K epoxide [94]. Both carboxylation and epoxidation reactions occur simultaneously. Vitamin K epoxide is then converted to vitamin K by VKOR [95,96,97]. Since vitamin K is continuously recycled within cells, vitamin K deficiency is uncommon in humans. The health benefits of vitamin K extend beyond coagulation to include hepatic functions [95]. More recently, vitamin K’s role in preventing and treating cancer [96] has been explored, as well as its implication in age-related diseases such as osteoporosis and osteoarthrosis, cardiovascular diseases, and neurodegenerative diseases [97,98,99]. 

##### B Vitamins 

B vitamins constitute a cluster of seven essential water-soluble vitamins; B_1_ (thiamine), B_2_ (riboflavin), B_3_ (niacin), B_5_ (pantothenic acid), B_6_ (pyridoxine), B_9_ (folate) and B_12_ (cobalamin). B vitamins are primarily produced within the mitochondria, chloroplasts, and the cytosol of plants, and play essential roles in energy production, and the composition and alteration of bioactive compounds through catabolic and anabolic processes, respectively [100]. In a significant portion of enzymatic processes, B vitamins carry out physiological functions by acting as coenzymes. As coenzymes, the biologically active forms of B vitamins tightly bind to the apoenzyme of a protein, producing a holoenzyme that is complete and catalytically active [101]. Through this binding activity, B vitamins play various ubiquitous roles in cellular functioning. The ubiquitous role of B vitamins is demonstrated by vitamin B_6_. Vitamin B_6_ functions primarily through its bioactive form, pyridoxal 5′-phosphate. Pyridoxal 5′-phosphate is an important cofactor that influences the functionality of several enzymes that are necessary for the production, degeneration, and conversion of amino acids in all organisms [101]. The essential requirement of B vitamins is also observed with coenzyme A (CoA), the bioactive coenzyme of vitamin B_5_. CoA is a compulsory co-factor for approximately 4% of mammalian enzymes functioning as a carbonyl-activating group and acyl carrier in numerous biochemical transformation reactions [102]. B vitamins may also act as precursors of metabolic substances, although this is less frequent. CoA also provides a good example of this. CoA can be further acetylated by acetyltransferase enzymes to produce acetyl-CoA, which participates in the biochemical metabolism of proteins, carbohydrates, and lipids, as well as energy production [103]. Notably, B vitamins possess various brain-specific functions. Vitamin B_1_ plays a critical role in the synthesis of amino acid precursors for neurotransmitters such as acetylcholine (Ach) and was reported to have neuromodulatory functions, including alterations to cholinergic transmission [104]. Vitamin B_1_ plays structural and functional roles within cell membranes, including in neurons and neuroglia [105] and vitamin B_1_ deficiency resulted in AD-like abnormalities, such as dysregulation of the cholinergic system, reduced neurotransmitter levels and memory deficits, in preclinical mouse models [106,107,108].

##### Vitamin C

Vitamin C (ascorbic acid) is predominately found within cells in its redox state, ascorbate [109]. Since humans are unable to endogenously produce vitamin C, the nutrient is obtained from fruit and vegetable sources such as citrus fruits (orange, berries, tomatoes) and leafy green vegetables (broccoli, brussels sprouts). Vitamin C primarily functions as a cofactor for many enzymes, such as hydroxylases, that are implicated in collagen synthesis [110]. More importantly, vitamin C acts as a potent antioxidant through attenuating lipid peroxidation. Lipid peroxidation is a form of radical chain reaction that is initiated by ROS-mediated dissociation of hydrogen atoms from C-H bonds producing lipid radicals [111]. ROS are often entrenched within lipid bilayers [111]. This renders lipids susceptible to the harmful effects of free radicals. Vitamin C can prevent lipid peroxidation by scavenging ROS and working synergistically with other antioxidant compounds, such as vitamin E, to reduce radical formation through the vitamin E redox cycle [112]. Vitamin C is also an exceptional source of electrons. Vitamin C can donate electrons to free radicals that seek out electrons from cellular components such as lipids, proteins, and DNA [111]. By donating an electron to free radicals, vitamin C stabilizes these volatile compounds, reducing their reactivity and the subsequent cellular damage. Vitamin C is constantly recycled within the cell, which increases its antioxidant activity [113]. As a donor of high-energy electrons, vitamin C is oxidized to dehydroascorbic acid. Dehydroascorbic acid can be converted back to vitamin C by dehydroascorbate reductase to be reused or further metabolized, which releases more electrons for ROS stabilization [110,111]. However, the ability of vitamin C to act as a reducing agent for metals such as Cu and Fe heightens the pro-oxidant composition and activity of these metals [114]. Therefore, vitamin C can behave as an antioxidant and a pro-oxidant, and this may depend on its concentration. The pro-oxidant role of vitamin C was initially reported to occur at low concentrations and the antioxidant activities at high concentrations [114]. However, more recent reports contradict these findings and suggest a switch-like behaviour of vitamin C, where it possesses bimodal activity as an antioxidant under normal conditions and switches over to a pro-oxidant at high concentrations and/or under pathophysiological conditions [115,116]. This raises questions regarding the therapeutic use of vitamin C when there is uncertainty of its antioxidant status within the literature [113,114]. Nonetheless, the potential health benefits of vitamin C continue to be explored for their therapeutic effectiveness in several diseases such as cancer, cardiovascular disease, diabetes, immunity, and neurodegenerative disorders [116,117,118,119].

Vitamin C assists in maintaining the function and integrity of various processes within the CNS including antioxidant protection, neuronal development and differentiation, myelination, catecholamine synthesis and regulation of neurotransmission [120]. Studies using animal models have reported detrimental impacts on the brain when vitamin C is decreased, such as enhanced oxidative stress, increased mortality, and the acceleration of amyloid plaque development and aggregation [121,122,123]. Vitamin C deficiency also reduced blood glucose levels and caused oxidative damage to proteins and lipids in the cortex of mice [122,124]. Dopamine and serotonin metabolites in the cortex and striatum of mice and physical strength and locomotor activity were decreased, and treatment with vitamin C was able to restore these deficits [125]. Since the highest concentrations of vitamin C are found in the brain [126] and several neurodegenerative diseases are characterized by oxidative stress, the protective role that vitamin C may play in altering the development and progression of neurological disease such as AD is being investigated.

##### Carotenoids

Carotenoids are a group of natural pigments that exist ubiquitously across all organisms [127]. Carotenoids perform active roles in photosynthesis in plants and function primarily through photoprotection in non-photosynthetic organisms [127]. In humans, carotenoids, which are found in blood and tissues, are essential precursors of vitamin A. Carotenoids maintain their status as antioxidants through their efficiency in chemically and physically quenching singlet oxygen and scavenging other ROS [127,128]. The structure of carotenoids is the most significant characteristic, contributing to their protective effects. Carotenoids are comprised of several conjugated double bonds, which are essential for photoprotection in all living organisms and light absorption in photosynthetic organisms [128]. Additionally, carotenoids are lipophilic compounds; therefore, they primarily reside within cell membranes. 

The most commonly described carotenoids include β-carotene, α-carotene, lutein, lycopene, and zeaxanthin. β-carotene and lycopene are examples of rigid hydrocarbons that are entirely organized within the inner portion of the lipid bilayer [129]. Lutein and zeaxanthin are polar compounds that contain oxygen atoms and exist horizontally to the membrane surface, exposing their hydrophilic segments to the aqueous surroundings [129,130]. It is suggested that the inclusion of carotenoids may impact membrane properties such as permeability, thickness, fluidity, and rigidity, all of which are essential for maintaining membrane integrity [131]. Membrane modifications by carotenoids may enhance resistance to ROS, thereby reducing susceptibility to ROS. Several reports have described the participation of carotenoids in various biological systems and general physiology. These include modulating gap junction communication via intracellular signaling pathways [131] and regulating cell cycle, differentiation, and growth factors [132]. Carotenoids and their metabolites have been implicated as having functions in human health and providing protection in various ROS-induced disorders. These roles include but are not limited to cognitive functions [133,134,135], cancer prevention [131,136], immune stimulation/modulation [137], fertility [138,139] and genomic impacts on transcription and translation [140].

##### Polyphenols

**Figure 2:**
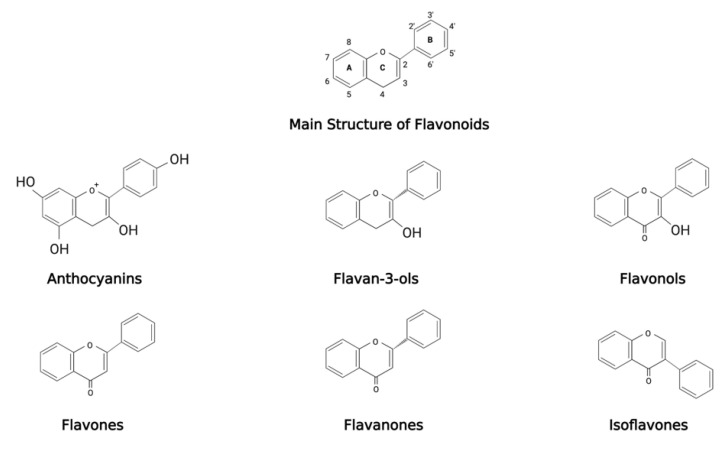
Chemical structure of flavonoid polyphenols. Created with BioRender.com. The flavonoid subclass of polyphenols is further classified into six main groups: anthocyanins, flavan-3-ols, flavonols, flavone, flavanones and isoflavones. Differences in chemical structure arise based on variations in the placement and number of hydroxyl groups and unsaturated bonds.

Polyphenols are naturally occurring compounds found in food sources such as beverages, cereals, fruits, and vegetables and are classified based on chemical structure and resulting activity. The primary classes of polyphenols are phenolic acids, flavonoids, lignans and stilbenes [141]. Phenolic acids are further divided into two classes: hydroxybenzoic acids and hydroxycinnamic acids. Hydroxybenzoic acids are less common than hydroxycinnamic acids and consist of compounds such as gallic and vanillic acid [142]. Hydroxycinnamic acids include caffeic, ferulic, ρ-coumaric and sinapic acids [142]. Flavonoids are the most highly studied cluster of polyphenols. Compounds within this group share a primary structure consisting of two aromatic rings, held by three carbon atoms creating an oxygenated heterocycle [143]. Flavonoids are separated into six subdivisions: anthocyanins, flavan-3-ols, flavonols, flavones, flavanones and isoflavones (Figure 2). Variations within these clusters are derived from differences in the composition and number of hydroxyl groups and their degree of glycosylation and/or alkylation [143]. The most widely studied flavonoids Are catechins, commonly found in green tea, quercetin, found in red wine and foods, and myricetin, which is also commonly found in medicinal plants [144,145,146]. Lignans are referred to as diphenolic compounds, formed by two cinnamic acid residues dimerizing to create a 2,3-dibenxylbutane structure. Many lignans, such as secoisolariciresinol, are regarded as phytoestrogens, possessing antioxidation and antitumor bioactivities [144]. Stilbenes are primarily found in grape skins and berries, and the most prevalent of this subdivision are resveratrol and its derivative pterostilbene [145]. Stilbenes, although low in the human diet, have been implicated for their potential for treating human disease due to their antioxidant and anti-inflammatory activities [145,146]. Other polyphenols include curcumin and gingerol, which have both been reported to provide health benefits and restoration to normal physiology in diseased states [146,147,148]. Overall, there is evidence to support the protective role of polyphenols in multiple disease conditions, such as cancer [149,150,151], cardiovascular disease [152], type 2 diabetes and obesity [149,153], inflammation [154] and neurodegenerative diseases [155,156,157]. Notable polyphenolic compounds studied for their neuroprotective effects include resveratrol, curcumin, quercetin, and epigallocatechin-3-gallate (EGCG) [158]. Recent studies examining cognitive deficits in transgenic AD mice report improvements in AD-like cognitive deficits through anti-amyloidogenic, anti-inflammatory and anti-apoptotic effects of polyphenolic compounds [159,160,161,162].

##### Minerals

Recently, minerals have been studied for their participation in the antioxidant defence system [163,164,165,166,167,168,169,170,171]. Cu is one of many trace elements that are essential to the biochemistry of active organisms due to its activity as a cofactor and a constituent of metalloenzymes [163]. Cu participates in electron transfer catalysis due to its ability to maintain two oxidation states [164]. Modest amounts of Cu are necessary and beneficial in maintaining metal homeostatic levels; however, the accumulation of redox transition metals such as excess Cu within tissues is cytotoxic [164]. Disturbances in metal homeostasis stimulate the development of oxidative stress and free radical formation, targeting membranes and essential molecules. At high levels, Cu has been implicated in the pathology of neurodegenerative conditions [163]. A study examining the effects of excess Cu showed significantly lower SOD and GSH activity in the brain tissue of Cu-intoxicated rats [163]. Similarly, Fe can result in comparable toxicity due to its ability to donate and accept electrons. Redox-active Fe is a significant contributor to oxidative damage in cellular compartments through the generation of free radicals from ROS by reducing H_2_O_2_ to produce ^•^OH radicals [165]. This eventually results in damage to several cellular structures, as the ^•^OH radical formed by Fe and even Cu can react with H_2_O_2_ to stimulate lipid peroxidation [166]. For this reason, Fe is predominantly bound to other molecules for transport and storage, leaving minuscule amounts of redox-active Fe in the labile pool. Even then, Fe does not remain unbound, as it forms complexes with carboxylates, phosphates, and peptides within the labile pool [166]. 

Mn is an essential element in the synthesis and activation of several enzymes. Its main antioxidant activity occurs through its role as a metalloenzyme via SOD2 to reduce mitochondrial oxidative stress [167]. SOD2 is the principal antioxidant that probes for O_2_^•−^, produced within the mitochondria to protect against oxidative stress [167]. SOD2 has also been suggested to provide protection for several disease states such as atherosclerosis, metabolic syndrome, and obesity [167]. Se is another natural trace element. Se is necessary for the composition of selenoproteins, which have been reported to play a critical role in the antioxidant defence system. The activity of GPx, one of the most efficient enzymatic antioxidants, is relatively dependent on Se [168]. Se is inorganically present as selenates, selenides, and selenite, which are more toxic than the organic states selenomethionine and selenocysteine [168]. Se present with GPx has been implicated in the repair of damaged DNA, and the ability to increase GPx activity contributes to the potential benefits of increased Se intake [169]. 

Zn has been considered an essential metal since its deficiency in humans was first recognized over 50 years ago [170,171]. Zn acts as an antioxidant through multiple mechanisms. Zn can compete with Cu and Fe for binding to proteins and cell membranes, which displaces redox-active Cu and Fe, resulting in the generation of ^•^OH from H_2_O_2_. Zn can also protect biomolecules such as sulfhydryl groups by binding to them, preventing oxidation [172]. Zn is also capable of enhancing the activation of antioxidant enzymes CAT, SOD and GSH, and diminishing the activity of pro-oxidant enzymes such as inducible ^•^NO synthase and NADPH oxide, while blocking the production of lipid peroxidation products [172]. Interestingly, Zn has been reported to upregulate nuclear erythroid 2-related factor 2 (Nrf2) activity, resulting in reduced oxidative stress [173,174]. Nrf2 is a member of the “cap’n’collar” subfamily of basic region leucine zipper transcription factors [175]. Nrf2 regulates the gene expression of many antioxidant and detoxifying enzymes such as SOD and GSH, and glutathione-S-transferase-1 and heme oxygenase-1, respectively. The binding activity of Nrf2 to the antioxidant response element found in the promoter of these target genes leads to the production of enzymes that function as part of the antioxidant defence system [176]. 

##### Estrogens

Neuroprotective antioxidants also include compounds such as hormones that are capable of exerting antioxidant effects within the body. Estrogens have been of particular interest as they are well-documented for their neuroprotective roles as steroid hormone antioxidants [177]. The neuroprotective effects of estrogen are proposed to occur through several mechanisms, including estrogen receptor (ER)-dependent and ER-independent actions, and several studies have explored the effects of estrogens in the aging brain and cognition. Estrogens are reportedly involved in learning and memory [178,179] and protect against neurodegenerative conditions such as AD [180,181]. Studies have explored the potential of estrogen replacement therapy (ERT) on improvements in cognitive function. These reports support the postulation of estrogen-mediated enhancements of cognitive function in women across age ranges and defer the onset of AD [182,183,184]. While estrogens have been reported to provide benefits against disease onset and progression, several findings contradict the role of estrogens in neurodegenerative diseases such as AD, reporting minimal differences in cognitive function between placebo and estrogen-treated groups [185,186,187]. These discrepancies leave room for further clarification and investigations into the therapeutic impacts of estrogens by means of ERT. Therefore, although it appears that estrogens may provide protective effects against the onset of AD, further clarification is required to determine whether estrogens are effective once neurodegenerative conditions have already developed.

**Figure 3:**
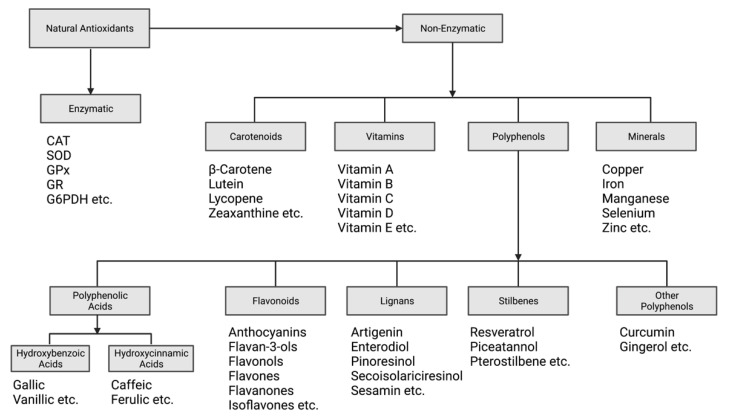
Schematic diagram of classification of natural antioxidants. Created with BioRender.com. CAT, catalase; SOD, superoxide dismutase; GPx, glutathione peroxidase; GR, glutathione reductase; G6PDH, glucose-6-phosphate dehydrogenase.

In summary, antioxidants function through three main mechanisms of action: they (1) act as scavengers to terminate or prevent the production of free radicals; (2) inhibit chain initiation and proliferation reactions; and (3) repair damaged DNA, proteins, and lipid biomolecules. A schematic representation of the classification of natural antioxidants is depicted in Figure 3.

## 3. Oxidative Stress in Alzheimer’s Disease

The role of oxidative stress in the pathogenesis of AD is well-established in the literature [188,189,190,191,192,193,194,195,196,197,198,199,200,201,202]. To this effect, the oxidative stress hypothesis of AD development postulates potential mechanisms by which oxidative damage causes and/or contributes to the development and progression of AD [203]. This hypothesis is supported by findings from molecular, genetic, and biochemical studies and highlights the detrimental role of ROS in AD onset and progression. Heightened levels of biomarkers of oxidative stress, impairments in the antioxidant defence system, gene mutations and mitochondrial dysfunction have all been implicated [194,196]. As previously mentioned, highly reactive molecules such as ROS target biomolecules such as DNA, proteins, and lipids and, in the instance of AD, mitochondrial dysfunction is proposed to underlie the increase in ROS [191,192,193,194,195,196,197,198,199,200,201,202,203]. Neuronal mitochondria consume high amounts of intracellular oxygen to perform essential functions including energy metabolism, the metabolism of amino acids, fatty acids and lipids, intracellular calcium homeostasis, ROS generation and regulation and more [204]. During mitochondrial respiration, O_2_^•−^, a by-product of adenosine triphosphate production, is created. In large amounts, O_2_^•−^ contributes to oxidative stress by oxidizing cellular targets directly or indirectly by reacting with other molecules and oxidants to form additional ROS and reactive nitrogen species [204]. Mitochondria also produce H_2_O_2,_ which can further exacerbate oxidative stress by the endogenous conversion reaction of H_2_O_2_ to ^•^OH by Fe^2+^ via the Fenton reaction [190,204]. Although the primary generation sites of O_2_^•−^ are mitochondrial respiratory transport chains I and III, additional cellular sources that could contribute to neuronal oxidative stress include xanthine oxidase, NADPH oxidase and cytochrome P450 enzymes. To prevent a cascade of ROS production, O_2_^•−^ is neutralized by SOD (Figure 1). Oxidative stress does not exist alone as a potential cause or contributing factor to AD [205,206,207,208,209]. Oxidative stress is reported to contribute to other hypotheses of AD, which implicate the aggregation of intracellular tau, elastin degradation, *N*-methyl-d-aspartate receptor (NMDAR)-mediated cell stress and abnormal extracellular amyloid accumulation as the primary cause [210,211,212,213,214,215,216,217,218,219,220,221].

The tau hypothesis of AD is widely described in the literature and explains the role of tau-induced neurotoxicity via abnormal hyperphosphorylation of the microtubule-associated protein, tau [222,223,224,225,226,227,228,229,230,231]. Under normal physiological conditions, tau proteins stabilize microtubules within healthy neurons, which maintains neuron morphology and facilitates the transport of enzymes and organelles along the cytoskeleton [224,225,226]. This action is regulated by the level of tau phosphorylation, which primarily depends on the balance between phosphorylation and dephosphorylation, resulting in the activity of various tau kinases and phosphatases, respectively [223,224,225]. Tau hyperphosphorylation reduces the tau microtubule-binding affinity, resulting in the destabilization of microtubules and oligomerization of hyperphosphorylated tau monomers [226,227,228]. In turn, tau oligomers aggregate to form neurofibrillary tangles that induce neurotoxicity and eventual cell death [229,230,231]. The link between oxidative stress and tauopathies is also well-described and attributes tauopathy to oxidative stress-induced aggregate formation that results in the degradation of the microtubule network [232,233,234,235,236].

A recent review by Atlante et al. [237] explains the active and reciprocal relationship between oxidative stress and tau pathology in AD. The researchers report both oxidative stress-induced tau phosphorylation and tau-induced oxidative stress as contributors to the development of AD due to factors, including reductions in cytoplasmic SOD1 and mitochondrial SOD2, which increases the profile of tau phosphorylation and the induction of mitochondrial dysfunction, resulting in H_2_O_2_ production by hippocampal tau phosphorylation, respectively [237,238,239,240]. In vitro, the inhibition of glutathione, which triggered mild oxidative stress, increased the levels of phosphorylated tau [234] and the oxidation of fatty acids stimulated tau polymerization [241]. Several reports demonstrate that oxidative stress-induced increases in metal ion redox potential also stimulate the upregulation of tau kinases [242,243,244,245,246,247,248,249,250]. Amyloid-beta (Aβ) is also implicated as a contributor to the cascade of molecular events that result in tau hyperphosphorylation and the inhibition of tau binding to microtubules by promoting glycogen synthase kinase 3 (GSK3) activation [251,252,253,254,255,256,257].

Recently, researchers have identified elastin degradation as a potential contributor to aging, oxidative stress and AD pathology [258,259,260,261,262,263,264,265,266,267]. Elastin is an essential protein that maintains the structural matrix of organs and tissues, including the skin, lungs, cartilage, and blood vessels [258,259,260]. Although elastin is structurally stable, it readily undergoes proteolytic degradation, producing elastin-derived peptides (EDPs) [261,262,263]. Utilizing in vivo and in vitro models, researchers have found that EDPs enhance Aβ formation, which could contribute to subsequent AD development [257,264]. Additionally, EDPs released from elastin due to proteolytic degradation gradually develop into amyloid-like structures [265,266]. Interestingly, like tau, described above, a recent review by Szychowski and Skóra examined the reciprocal relationship between the production of ROS and EDPs [268]. An essential factor in the mechanism of action of EDPs is the peroxisome proliferator-activated receptor gamma (PPARγ) pathway, which is reported to increase the production of ROS by increasing calcium (Ca^2+^) influx and disrupting the activity and expression of antioxidant enzymes [268,269,270]. PPARγ is reported to enhance SOD, CAT and GPx activity and increase lipid peroxidation [271,272]. Resveratrol, a PPAR agonist, is an example of an antioxidant compound that exerts these neuroprotective properties and was examined for its therapeutic potential in AD [273,274]. Interestingly, in other forms of neurodegeneration, such as ischemia, EDPs are formed in the brain post-injury, which is also when Aβ formation is induced [275]. Some reports have demonstrated EDP-induced increases in ROS in the brain in neuronal stem cells as well as astrocytes [270,272,276]. In turn, EDPs are reported to induce Aβ formation by inducing the overexpression of γ-secretase, which results in excess cleavage activities and the overproduction of Aβ [265]. A few studies suggest that proteoglycans, which are present in the extracellular matrix, may contribute to AD pathogenesis by promoting the fibrilization of Aβ and tau and protecting Aβ from proteolytic degradation [277,278]. The presence and accumulation of EDPs in the brain are also associated with age and correspond with the incidence of AD [275,279]. Increased levels of EDPs have also been detected in CSF patients following a stroke [275,280]. Taken together, these findings suggest the presence of EDPs as potential biomarkers of neurodegenerative disease and that therapies directed at elastin degradation may be useful in the treatment of AD.

The NMDAR hypothesis suggests that excess NMDAR activation leads to the neurodegeneration that occurs in AD [281,282]. Under normal conditions, excitatory neurotransmission by glutamate through the NMDAR is essential for synaptic plasticity and the survival of neurons [281,282]. However, the superfluous activity of the NMDAR induces excitotoxicity causing neuron death, which is a foundational mechanism of the neurodegeneration observed in AD [281,282]. Since the NMDAR mediates Ca^2+^ regulation and influx [283,284,285,286], dysregulation and overactivity are reported to induce oxidative stress by enhancing the production of ROS within the brain through mechanisms involving EDPs [287,288]. Ca^2+^ channel blockers such as nifedipine and verapamil are also reported to attenuate ROS production induced by EDP fragments, influence EDP levels in the brain and possibly delay the progression of AD [276,289,290].

Several reports support the involvement of oxidative stress in Aβ toxicity involving metal ions [291,292,293,294,295,296,297,298,299,300,301,302,303,304,305]. Extracellular senile plaques/fibrils comprised of aggregated Aβ peptides exist with metal ions such as Fe, Cu and Zn [293,294,295]. These redox-active metal ions can catalyze the production of ROS when bound to Aβ [296,297,298,299,300,301,302,303,304,305]. Subsequently, newly generated ROS can oxidize both Aβ peptides as well as surrounding biomolecules such as lipids, nucleic acids, and proteins [306,307,308,309,310,311,312,313,314,315]. The oxidation of lipids such as cholesterol within neuronal membranes obstructs membrane integrity [313,314]. In addition, the oxidation of Aβ by ROS and redox-active metal ions impairs its clearance by low-density lipoprotein receptor-related proteins, which could contribute to the accumulation of Aβ in AD [315,316]. 

Oxidative stress, caused by the production of ROS, creates a favourable environment for Aβ synthesis and accumulation through transcriptional, translational, and epigenetic mechanisms [317,318,319,320,321,322,323,324,325,326,327,328]. Researchers have found that the activation of stress-related signalling pathways stimulates the transcription of amyloid precursor protein (APP) and beta-secretase 1 (BACE1), an essential enzyme for Aβ production [318,319,320]. Additionally, an enhanced protein expression of BACE1 due to ROS such as H_2_O_2_ has been reported and is proposed to be regulated by eukaryotic translational initiation factor-2alpha (eIF2α), which was implicated in AD when activated via phosphorylation [321,322]. Several studies have established the role of epigenetic modifications, such as DNA methylation, histone acetylation and chromatin remodelling, in changes to Aβ and AD progression [323,324,325,326,327]. More recently, researchers have demonstrated a link between oxidative stress and epigenetic changes in Aβ production. Gu et al. showed that when neuroblastoma cells were treated with H_2_O_2_, a significant decrease in DNA methylation and an increase in histone acetylation occurred [328]. This resulted in increased APP and BACE1 transcription, which was followed by enhanced Aβ production and plaque accumulation [328]. 

In turn, Aβ exerts its toxic effects through several mechanisms, including oxidative stress. Aβ has been reported to alter mitochondrial function by localizing within the mitochondrial membrane, where it blocks the transport of nuclear-encoded mitochondrial proteins [236,237,238,239,240,241,242,243,244,245,246,247,248,249,250,251,252,253,254,255,256,257,258,259,260,261,262,263,264,265,266,267,268,269,270,271,272,273,274,275,276,277,278,279,280,281,282,283,284,285,286,287,288,289,290,291,292,293,294,295,296,297,298,299,300,301,302,303,304,305,306,307,308,309,310,311,312,313,314,315,316,317,318,319,320,321,322,323,324,325,326,327,328,329]. Additionally, Aβ prevents normal neuronal functions by interacting with mitochondrial proteins, dysregulating the electron transport chain, and stimulating the production of ROS [237,238,239,240,241,242,243,244,245,246,247,248,249,250,251,252,253,254,255,256,257,258,259,260,261,262,263,264,265,266,267,268,269,270,271,272,273,274,275,276,277,278,279,280,281,282,283,284,285,286,287,288,289,290,291,292,293,294,295,296,297,298,299,300,301,302,303,304,305,306,307,308,309,310,311,312,313,314,315,316,317,318,319,320,321,322,323,324,325,326,327,328,329,330,331,332,333,334,335,336,337,338,339]. Additional actions include Aβ-mediated dysregulation of Ca^2+^ homeostasis, ion leakage through pore formation and depletion of membrane potential [340,341,342]. As a result, this disrupts the cytoskeleton, causes synaptic dysfunction, and stimulates neuronal apoptosis [343]. The examination of human brains from patients diagnosed with AD showed a high degree of membrane damage due to oxidation within the cerebral cortex [344]. Evidence supports the validation of the oxidation of proteins as biomarkers of AD, as enhanced levels of carboxylate proteins have been reported in the hippocampus and parietal cortex [345,346,347]. 

Considering that ROS production can be a product of tissue injury [348,349,350], it is currently unclear whether oxidative stress is a primary or secondary cause of AD. Even as a secondary cause, oxidative stress perpetuates a detrimental cascade of toxic events that ultimately result in neuron loss. Despite profound evidence supporting the role of oxidative stress in the pathogenesis and progression of AD, none of the currently available treatment options are designed to address oxidative stress. The development of innovative therapies that target the pathological contributors of the disease, such as ROS, could substantially improve the care of patients with AD.

## 4. Current Treatments for Alzheimer’s Disease

Currently, the only United States Food and Drug Administration (FDA)- and Health Canada-approved medications for AD fall under the classifications of acetylcholinesterase (AChE) inhibitors and NMDAR antagonists [351,352,353]. Donepezil, galantamine and rivastigmine fall under the category of cholinesterase inhibitors. AChE is found predominantly in neuromuscular junctions and synapses of cholinergic neurons in the periphery and CNS, where it rapidly degrades ACh. This neurotransmitter is reported to be involved in learning and memory [354], and the loss of cholinergic neurons projecting from the basal forebrain to the hippocampus and cortex increases as AD progresses [355]. Donepezil and galantamine act by reversibly binding to AChE, which inhibits the hydrolysis (breakdown) of ACh, increasing its levels at synapses throughout the CNS [356]. Rivastigmine also acts to enhance cholinergic communication by binding to and inhibiting AChE, as well as butyrylcholinesterase [357]. These drugs are indicated as long-term symptomatic treatments of AD; however, these drugs lose their efficacy as fewer cholinergic neurons remain in the brain as AD progresses [355]. Donepezil is approved for all stages of AD, while rivastigmine and galantamine are recommended for patients exhibiting mild to moderate symptoms [358]. Memantine, an NMDAR antagonist, acts by blocking the flow of ions through the NMDAR ion channel [359]. Memantine is indicated for moderate to severe AD [360]. Manufactured conjugate (combination) drugs, comprised of donepezil and memantine as extended-release capsules, also exist to alleviate the pill burden of taking multiple medications and increase patient compliance, while mitigating challenges with swallowing that are often associated with AD [361].

Non-pharmacological treatment options include identifying any potential harmful supplements and medications and removing them from the patient’s regimen [362]. First-line treatments for the neuropsychiatric symptoms and behavioural issues associated with the disease include repetitive evaluations, identifying triggers, providing psychoeducation, and modifying both behavioural and environmental interventions [362,363].

The currently available treatments for AD are ineffective in preventing, delaying progression, or curing disease [351]. This necessitates the development of novel disease-modifying therapies that target the pathological hallmarks of the disease, such as tau protein hyperphosphorylation, the development and accumulation of Aβ, inflammation, and oxidative stress [351]. Recently, aducanumab, the only potential disease-modifying therapy, was approved by the FDA through the FDA’s accelerated approval program. Aducanumab is a human monoclonal antibody that significantly reduced the formation and increased the clearance of existing Aβ plaques in mouse models of AD [364,365,366]. However, there is controversy regarding whether the drug slows disease progression in humans, as findings from currently available clinical trial data indicate strategies that reduce amyloid levels do not significantly improve cognition [367]. Biogen, the drug company that created aducanumab, is conducting additional studies to assess the clinical benefit of aducanumab post-approval. If the additional studies fail to show evidence of a clinical benefit, the FDA can withdraw drug approval. Phase 4 clinical trial results for aducanumab are expected to be accessible as early as 2030. 

Despite tremendous efforts to find a cure or an effective treatment, AD remains progressive and incurable. Studies utilizing animal models to depict AD show improvements in AD-like phenotypes when utilizing novel therapies such as antioxidants [368,369,370]. Some epidemiological studies also show a reduced risk of AD due to the dietary intake of antioxidants [371,372,373]. Recently, researchers have begun exploring the use of antioxidants in combination with other antioxidant compounds, as well as drugs that are currently being used to treat neurogenerative diseases. This is referred to as combination or conjugate therapy.

## 5. Conjugate Therapies and the Blood Brain Barrier

The concept of conjugate drug therapy was initially developed as a novel avenue for cancer treatment and has now produced treatment strategies such as antibody–drug conjugates (ADCs). ADCs are designed to target and destroy cancer cells while preserving healthy cells by chemically linking two or more distinct substances [374]. ADCs utilize monoclonal antibodies to deliver cytotoxic agents to antigen-expressing target cells. This approach to treatment has been applied to various types of cancer, such as breast cancer, non-small-cell lung cancer and ovarian cancer, to name a few [375,376,377]. The impact of oxidative stress as a contributing factor to the development of various cancers is well-studied [378,379,380,381,382,383,384], which makes it a primary target in the development of anti-cancer drugs. Interestingly, researchers also utilize the harmful effects of ROS as a tool to target cancer cells [385,386,387,388,389,390]. This includes activating ROS-specific cell death mechanisms such as apoptosis, autophagy, ferroptosis (Fe-dependent) and necrotic cell death in tumour targeted therapy [385,386,387,388,389,390]. An example of the application of ROS in cancer therapy is through targeted tyrosine therapies, which include monoclonal antibodies and small-molecule inhibitors that have been shown to elicit anticancer ROS-mediated effects [391,392,393,394,395,396]. More recently, ADCs have been applied to neurological cancers and neurodegenerative diseases. One example is glioblastoma, an aggressive form of brain cancer that can develop within the brain and spinal cord [397]. However, the efficacy and applications of ADCs within the CNS have been reported to be limited due to the inability of these large drug conjugates to cross the blood–brain barrier (BBB) [398].

When developing novel therapies for neurodegenerative disorders, several factors must be considered for effective drug delivery. One of the most significant is BBB permeability. Unfortunately, the effectiveness of various antioxidants, alone or in conjugated form, are limited by their inability to cross the BBB [399]. The BBB functions as the brain’s endogenous defence system, by excluding non-lipophilic and high-molecular-weight compounds. For a drug or compound to elicit its desired effects, it must first permeate the BBB to reach its drug targets. BBB permeation can occur through several mechanisms, including transmembrane diffusion, saturable transporters, absorption via endocytosis and other extracellular pathways [399]. Several drugs cross the BBB through transmembrane diffusion. This mechanism largely depends on the drug or compound’s ability to cross the cell membrane, which depends on the exogenous compound’s molecular weight, charge, and degree of lipid solubility [400]. Once a drug or compound has diffused through the lipid membranes of the BBB, it will enter the brain’s aqueous environment before reaching its therapeutic target. Therefore, the substance must possess a desirable level of lipid solubility but not be “too lipid soluble”, so that it does not get trapped within the BBB [399]. Saturable transport systems are also an advantageous mechanism of drug delivery and transport across the BBB. Transporters increase the rate of uptake across the BBB compared to what a drug would achieve through transmembrane diffusion alone [401]. However, uptake is limited, as transport occurs via saturable transport systems [401]. The BBB also contains transporters that remove compounds from the brain. These transporters assist with removing toxins from the brain but can also reduce the effectiveness of some therapeutics by increasing their efflux [402]. Under normal conditions, BBB uptake and efflux transporters adapt to meet the needs of the CNS; however, during diseased states, dysregulation can occur. This is observed in AD, for example. Deposition of Aβ damages the BBB and, inversely, reduces Aβ efflux, which contributes to the disease cyclically, as disturbances in BBB function further provoke Aβ deposition [403,404]. BBB dysregulation can be further exacerbated by oxidative stress, either directly or by stimulating the damaging effects of the Aβ peptide (discussed above).

Several antioxidants, including non-traditional antioxidants such as ebselen, have been explored for their ability to cross the BBB and exert neuroprotective roles within the brain when administered alone, in combination, or conjugated with other compounds. Ebselen, a Se-containing compound, has also been assessed for its GPx-like effects [405,406]. As previously mentioned, Se is an essential trace element that maintains antioxidant activity within the brain through oxidative stress resistance [407]. Ebselen has been shown to mitigate the impacts of AD pathology in cell line and primary culture models, as well as triple transgenic AD mouse models. A study conducted by Xie et al. demonstrated the ability of ebselen to inhibit oxidative stress in both cellular and mouse models of AD through enhancing GPx and SOD activity while reducing the activity of p38 mitogen-activated protein kinases [408]. Additionally, ebselen was able to reduce oligomeric Aβ levels within the brains of AD mice by diminishing the expression of APP and BACE-1, both of which are involved in the amyloidogenic pathway of Aβ synthesis [408]. Similar mechanisms have also been reported for other antioxidant compounds that have shown promise in studies utilizing animal subjects to model the onset and course of progression of AD, and the impacts of novel drugs and/or compounds as therapeutic options. Antioxidants that have been explored in combination with other compounds in both cellular and animal models of AD including but not limited to ebselen and donepezil [409], lipoic acid and donepezil [410], ferulic acid and tacrine (the first AChE inhibitor approved for AD, but now discontinued) [411], and polyphenolic hybrids [412,413].

Researchers are currently exploring the neuroprotective roles of antioxidants in humans when these drugs are administered alone, in combination with other antioxidants or drugs but not chemically linked, or in conjugated form with other antioxidants or drugs. To be effective, potential antioxidant drug compounds must be lipid-soluble, small molecule, and/or be chauffeured by other mobilizing non-toxic substances from the bloodstream, through the BBB and into the brain. Instead of utilizing only one antioxidant compound, a combination of antioxidant compounds would increase the overall antioxidant capacity of the drug therapy, heighten the bioavailability to various cellular locations and increase the functionality of antioxidant molecules, such as through facilitating redox cycling [414].

## 6. Clinical Trials

Various studies have explored the role of compounds with antioxidant activity for the prevention and treatment of cognitive decline and dementia caused by AD. Table 2 summarizes the data from human clinical trials investigating antioxidants in AD. This summary includes results published within the last two decades and those available from on-going clinical trials. Data from clinical trials were collected from the NIH U.S National Library of Medicine site: ClinicalTrials.gov. The inclusion criteria for the clinical trials for this review required that the study (1) includes participants diagnosed with a neurodegenerative disease with evidence of oxidative stress such as AD, (2) utilized at least one natural antioxidant as a form of treatment or preventative therapy, (3) utilized more than one natural antioxidant as combination treatment, and/or (4) utilized a natural antioxidant in combination with a drug currently used to treat AD.

### 6.1. Vitamins

Studies have reported that vitamins may delay the progression of AD in patients with moderate to severe AD [435,436,437]. Notably, due to the findings from preclinical data supporting the potent effects of vitamin E [438,439,440,441,442], it has been explored as a suitable antioxidant treatment in humans. In addition, vitamin E has been tested in combination with other vitamins and drugs that are currently being used to treat AD for its neuroprotective effects. A randomized, controlled, double-blind study compared the effects of daily administration of 2000 IU vitamin E or 10 mg selegiline, administered alone or in combination, compared to a placebo over 2 years in 341 patients with moderate AD [415]. Selegiline is a selective irreversible monoamine oxidase B (MAO-B) inhibitor that increases the level of dopamine within the synapse by inhibiting dopamine metabolism and is primarily indicated for the treatment of PD [443]. However, earlier trials showed promise for its role in treating AD [444,445], which led researchers to explore the potential benefit of selegiline when combined with other promising compounds such as vitamin E. Based on the primary outcome measures from this study, including the time until death, institutionalization, loss of ability to perform activities of daily living, or severe dementia, this study reported that treatment with both vitamin E and selegiline slowed the progression of disease in patients with moderately severe impairment from AD [444]. The effects of vitamin E have also been examined compared to the AD drug donepezil [416]. However, this study, which included 790 patients with mild cognitive impairment (MCI) and probable AD, showed findings that conflict with other clinical trials involving vitamin E. Patients received 2000 IU vitamin E, 10 mg donepezil or placebo, daily for 3 years. The main findings from this double-blind study showed that vitamin E had no benefit, while donepezil was associated with a lower rate of progression in the first 12 months [416]. In contrast, patients with mild to moderate AD receiving 2000 IU vitamin E, 20 mg memantine or both daily for 5 years showed improvements compared to placebo in another randomized clinical trial [417]. Although a daily dose of 2000 IU vitamin E alone slowed functional decline, there was no difference between the groups receiving memantine alone or with vitamin E [417]. Vitamin E was also tested in combination with other vitamins and minerals [434]. A randomized control trial assessed the changes in cerebrospinal fluid (CSF) biomarkers related to AD and oxidative stress, cognition, and function after antioxidant administration in 75 patients with mild to moderate AD who received 800 IU vitamin E in combination with 500 mg vitamin C, 900 mg ALA and 400 mg coenzyme Q10 3 times/day or placebo daily for 16 weeks. The researchers found that these antioxidants did not influence CSF biomarkers related to amyloid or tau pathology. Although markers of oxidative stress in the brain were reduced, the researchers raised concerns that this antioxidant combination may promote cognitive decline, which would have to be assessed on a long-term basis [418].

B vitamins have also been investigated for their potential protective role in AD in human clinical trials. Although several studies are still ongoing, B vitamins are being explored for their impacts on factors such as changes in phosphorylated tau, brain energy metabolism, oxidative stress, and cognitive function [446,447]. One randomized clinical trial assessed the role of high-dose vitamin B supplementation on homocysteine levels among 340 patients with mild to moderate AD [419]. Elevated homocysteine levels are reported to be a risk factor for dementias such as AD and are attenuated by B vitamin supplementation [448,449,450,451,452]. In this study, patients received a combination of 5 mg folate, 25 mg vitamin B6 and 1 mg vitamin B12 or a placebo, daily for 18 months with the objective of assessing changes in the cognitive subscale of the Alzheimer’s Disease Assessment Scale (ADAS-Cog) [419]. The results indicated that although high-dose B vitamin supplementation reduced homocysteine levels, it did not slow cognitive decline in individuals with mild to moderate AD. The researchers note that several factors could have influenced this negative result. One of them is a difference in the reduction of homocysteine levels observed in participants with milder AD symptoms compared to those with moderate AD, which may indicate a need for further studies that separate the cohorts based on stage, as supplementation may be more beneficial in older individuals with higher homocysteine levels [419]. Additionally, factors such as mental health, diet, supplements, and the activity of the patients should be considered when monitoring or assessing cognitive decline in patients with AD as they may contribute to the worsening of symptoms over time. 

Vitamin D has also been explored, notably in combination with memantine [420]. Although this study is ongoing, researchers have set the criteria to assess the effects of vitamin D and memantine in 90 patients with moderate AD. Patients will receive 100,000 IU vitamin D_3_ (every 4 weeks), in combination with 20 mg memantine or placebo, daily for 24 weeks. The primary objective is to measure changes in cognitive performance measured with the ADAS-Cog and Mini-Mental State Examination (MMSE). Additional measures include changes in functional performance, posture and gait, and comparisons of compliance and tolerance to treatment. 

A multivitamin approach was also explored as a treatment for AD [421,453]. In one study including 135 patients with AD or MCI, patients received a multivitamin in the form of a nutraceutical formulation (NF) of 400 ug folic acid, 6 ug vitamin B12, 30 UI vitamin E, 400 mg S-adenosyl methionine, 600 mg *N*-acetyl cysteine, 500 mg, and acetyl-l-carnitine, daily for 1 year [421]. The primary outcome measures were cognitive improvement or maintenance of cognitive performance, rated by caregivers using the Dementia Rating Scale (DMS), CLOX-1 clock drawing test and the Neuropsychiatric Inventory Questionnaire (NPI-Q). The phase II study reported that patients who received the NF showed improvements compared to the placebo cohort, demonstrating a maintained or improved cognitive performance and mood/behaviour based on the DMS and CLOX-1 measurements. However, no significant improvements were reported in NPI-Q scores. These findings support the conclusions of the phase I study that reported maintenance and/or improvements in cognitive performance and mood/behaviour [453].

### 6.2. Polyphenols

Polyphenolic compounds are also being explored for their potential as antioxidant treatments for AD. Resveratrol, a potent stilbene antioxidant, has been assessed for its safety, tolerability and efficacy and its role in impacting biomarkers associated with AD. A pilot study involving 39 patients with mild to moderate AD showed that low-dose resveratrol was as safe and well-tolerated as a placebo when administered at 5 mg resveratrol in combination with 5 mg dextrose and 5 mg malate twice daily for 1 year [422]. However, a larger study was necessary to evaluate its beneficial effects. Another study that included 119 patients with mild to moderate AD receiving up to 1 mg resveratrol twice daily or placebo for 52 weeks demonstrated that resveratrol decreased CSF biomarkers, modulated neuro-inflammation, and induced adaptive immunity, which are linked to the progression of AD [423]. 

Curcumin is another polyphenol that showed promise in preclinical studies. However, these data lack translation in human clinical trials. Although human clinical trials are ongoing, some studies report that curcumin may not be as beneficial for the treatment of AD as some researchers had hoped. One study that included 36 patients with mild to moderate AD who were given 2 g or 4 g curcumin or placebo, daily for 24 weeks showed that although curcumin was well-tolerated, there was no clinical or biochemical evidence of efficacy [424]. In addition, the data suggested the limited bioavailability of curcumin [424]. This is supported by other reports describing fewer adverse events but a concern of elevated serum Aβ in patients receiving either 1 g curcumin or 4 g curcumin in combination with 120 mg ginkgo leaf extract daily for 6 months when compared to a placebo [425]. However, it is possible that this finding was the result of the other compounds consumed in combination with curcumin. 

Quercetin and EGCG are also popular antioxidants that are currently under investigation in ongoing trials recruiting patients with early AD and/or patients that are carriers of the ApoE4 allele [426,427,428,429,454]. Carriers of the ApoE4 allele present an increased susceptibility and risk of developing AD [449]. One ongoing study is exploring the role of quercetin on changes in cellular senescence blood markers in patients with early AD [426]. Patients will receive a combination of 1000 mg quercetin and 100 mg dasatinib, a tyrosine receptor inhibitor, or placebo for 2 consecutive days followed by a 13-day +/− no drug period for 12 weeks. Another ongoing study is examining the cognitive impacts of EGCG treatment in patients with early AD [428]. Patients will receive daily treatments of tri-monthly increasing doses of 200 mg, 400 mg, 600 mg, and 800 mg EGCG or placebo for 18 months. Cognitive improvements will be assessed using the ADAS-Cog scale. 

Genistein, an isoflavone, has also been investigated both alone and in combination with other compounds for its potential neuroprotective effects in AD [430,431]. Ongoing trials are evaluating genistein-induced changes in Aβ concentrations in the CSF of patients with mild AD [430]. Genistein has also been tested for its effects on cognition in combination with other soy isoflavones such as daidzein [431]. A study of 72 patients with mild AD who received 100 mg of soy isoflavones or placebo daily for 6 months was tested for cognitive outcomes [431]. The 100 mg soy isoflavones combination consisted of genistein and daidzein in equal 50 mg capsules. Cognitive outcomes included language execution function, verbal memory and recall, attention, visual memory, and planning. However, the main findings from this study demonstrated that, after 6 months, the combination treatment of isoflavones genistein and daidzein did not benefit cognition in older men and women with AD [431]. This study was one of the first to examine the function of soy isoflavones in older adults with cognitive decline and AD. The researchers propose that these findings are likely influenced by individual differences in isoflavone metabolism. 

ALA has been explored in combination with omega fatty acids [432]. In a pilot trial, 39 patients with mild AD received 600 mg ALA and 3 g fish oil, 3 g fish oil only or placebo daily for 12 months. The results showed that the combination of ALA with omega-3 fatty acids slowed cognitive and functional decline compared to placebo. However, since the size of study participants is relatively small, future studies that include larger sample sizes are essential to determine the neuroprotective benefits of this antioxidant combination as a treatment for AD.

### 6.3. Minerals

Minerals such as Cu and Se have also been explored for their role in AD and potential to act as a form of therapy. An ongoing trial is evaluating the role of Cu on cognitive function in patients with mild to moderate AD [433]. Patients will receive 8 mg of Cu or placebo daily for 1 year. Changes in cognitive function will be measured using the ADAS-Cog scale. Se has also been studied for its potential role in AD when administered in combination with vitamin E [434]. The Prevention of Alzheimer’s Disease by Vitamin E and Selenium (PREADVISE) trial recruited 7540 men, of which 3786 participated and received 200 μg Se in combination with 400 IU Vitamin E, 200 μg Se + placebo, or 400 IU vitamin E + placebo or placebo + placebo, daily for 7–12 years [434]. The primary outcome was to assess the incidence of dementia (including AD); however, findings showed that neither supplement prevented the development of dementia or the progression from mild cognitive impairment to AD. 

Conflicting clinical trial data perpetuate the ongoing disposition on the benefits of antioxidants in AD treatment. Researchers have postulated that these disparities may be due to a variety of factors. Firstly, the equilibrium status between the production of oxidants and the presence of antioxidants is relatively unknown, and this creates a greater challenge when testing human subjects that may present remarkably different profiles of adequacy in endogenous antioxidant defence [455]. Secondly, factors such as the insufficiency of utilizing only one antioxidant compound in a treatment plan should be considered in addition to correcting for the dosages that would provide the most desirable effects specific to the patient [456,457]. Another factor, and arguably the most significant, is BBB permeability. There may be individual differences in BBB permeability; however, results from animal studies indicate that several antioxidants, alone and in combination, can permeate the BBB to some degree [458,459,460,461,462,463]. More recently, novel avenues for drug delivery have emerged to tackle this challenge. These include the utilization of nanoparticles as a strategy to deliver drugs into the CNS [464], as well as synthesizing antioxidant compounds that are chemically linked and developed to meet the criteria for BBB permeation [464,465,466]. Therefore, it is reasonable to conclude that the use of conjugated antioxidants, establishing a reliable profile of biomarkers for each patient and addressing BBB permeation may result in more conclusive findings regarding the effects of antioxidant therapy in AD.

## 7. Conclusions

AD is currently the leading cause of dementia worldwide, with a prevalence of more than 20 million, which is expected to double by 2040 [286]. Although research has progressed in investigating the etiology and pathogenesis of the disease, much remains unknown about AD. This review describes the well-documented role of oxidative stress in AD; however, the ability of antioxidants to prevent and/or mitigate the impacts of oxidative stress in AD remains uncertain. Compounds such as vitamins, carotenoids, polyphenols, and minerals have shown promise in cellular and animal-based models of AD, prompting their investigation in human clinical trials for their neuroprotective effects, both alone and in combination with other antioxidants or drugs that are currently approved for the treatment of AD. In general, results from previous and ongoing clinical trials remain inconclusive.

Although antioxidants show promise as potential therapies for AD, limitations exist regarding their capacity to treat AD. These limitations include challenges with dosing and determining appropriate timepoints and intervals for intervention, the probability that factors other than oxidative stress may be the predominant cause or propagator of neurodegeneration or that one antioxidant compound may not sufficiently combat oxidative stress to have an impact on disease development or progression. The latter consideration supports the need to explore the use of combination and/or conjugate antioxidant therapy where more than one antioxidant is utilized as a novel approach to treating AD and other neurogenerative conditions that include oxidative stress as a contributing factor. Other considerations for the development of therapies that target ROS-mediated harm in AD, include employing strategies that enhance the activity of molecular targets such as Nrf2 to increase the production of antioxidant enzymes and strengthen the endogenous antioxidant defence system. These approaches will enhance the understanding and application of antioxidant therapies in ROS-mediated neurodegenerative disease.

## Figures and Tables

**Table 1:** Some neurodegenerative diseases and conditions associated with oxidative stress.

Disease or Condition
Alzheimer’s disease [4,5]
Amyotrophic lateral sclerosis [6,7]
Corticobasal degeneration [8]
Creutzfeldt-Jakob disease (Prion disease) [9]
Down syndrome [10]
Diabetic neuropathy [11,12]
Friedreich’s ataxia [13]
Huntington’s disease [14,15,16]
Lewy body disease [17]
Multiple sclerosis [18,19]
Neiman-Pick C disease [20,21]
Neuromyelitis optica [22]
Parkinson’s disease [23,24,25]
Progressive supranuclear palsy [26]
Spinocerebellar ataxia [27,28,29]
Stroke [30,31,32]
Traumatic brain injury [33,34,35]

**Table 2:** Summary of clinical trials utilizing combination/conjugate antioxidants as preventative therapy or treatment in AD. ADCS-ADL: Alzheimer’s Disease Cooperative Study—Activity of Daily Living; ADAS-Cog: Alzheimer’s Disease Assessment Scale (Cog: Cognitive score).

Classification	Compound(s)	Participants	Intervention	Primary Outcome Measures	Main Results	In-Text Reference
Vitamins	Vitamin E + Selegiline	341 patients with moderate AD	2000 IU vitamin E, 10 mg selegiline, both or placebo daily for 2 years	Time until occurrence of death, institutionalization, loss of ability to perform activities of daily living, or severe dementia	Treatment with vitamin E or selegiline slowed the progression of disease in patients with moderately severe impairment from AD	[415]
	Vitamin E + Donepezil	790 patients with mild cognitive impairment (MCI)	2000 IU vitamin E, 10 mg donepezil or placebo, daily for 3 years	Clinically possible or probable AD	Vitamin E had no benefit. Donepezil was associated with a lower rate of progression in first 12 months	[416]
	Vitamin E + Memantine	613 patients with mild to moderate AD	2000 IU vita-min E, 20 mg memantine, both or placebo daily for 5 years	ADCS-ADL	2000 IU/day of vitamin E compared to placebo slowed functional decline. No difference between groups receiving memantine alone or memantine + vitamin E	[417]
	Vitamin E + Vitamin C + Alpha-Lipoic Acid	75 patients with mild to moderate AD	800 IU vitamin E + 500 mg vitamin C + 900 mg alpha-lipoic acid, 400 mg coenzyme Q10 3 times/day or placebo daily for 16 weeks	Changes in cerebral spinal fluid (CSF) biomarkers related to AD and oxidative stress, cognition and function	Antioxidants did not influence CSF biomarkers related to amyloid or tau pathology	[418]
	B Vitamins	340 patients with mild to moderate AD	5 mg folate + 25 mg vitamin B_6_ + 1 mg vitamin B_12_ or placebo daily for 18 months	Changes in the cognitive subscale of the ADAS-Cog	Regimen of high-dose B vitamin supplements does not slow cognitive decline in individuals with mild to moderate AD	[419]
	Vitamin D + Memantine	90 patients with moderate AD	100,000 IU vitamin D_3_ (every 4 weeks) + 20 mg memantine or placebo daily for 24 weeks	Change of cognitive performance	Ongoing	[420]
	Multivitamin	135 patients with AD or MCI	Nutraceutical formulation (NF) of: 400 ug folic acid, 6 ug vitamin B_12_, 30 IU vitamin E, 400 mg S-adenosylmethionine, 600 mg *N*-acetyl cysteine, 500 mg acetyl-l-carnitine daily for 1 year	Cognitive improvement or maintenance of cognitive performance	NF maintained or improved cognitive performance and mood/behaviour	[421]
Polyphenols	Resveratrol	39 patients with mild to moderate AD	5 mg resveratrol + 5 mg dextrose + 5 mg malate or placebo twice daily for 1 year	Evaluate the safety, tolerability and efficacy of resveratrol, glucose and malate in slowing the progression of AD	Low-dose resveratrol is safe and well-tolerated	[422]
	Resveratrol	119 patients with mild to moderate AD	Up to 1 mg resveratrol twice daily or placebo for 52 weeks	Safety and tolerability of treatment with resveratrol and change in ADL	Resveratrol decreases CSF biomarkers, modulates neuro-inflammation and induces adaptive immunity	[423]
	Curcumin	36 patients with mild to moderate AD	2 g or 4 g curcumin or placebo daily for 24 weeks	Examine safety and tolerability of curcumin and determine its side effects on patients	Curcumin well-tolerated. Unable to demonstrate clinical or biochemical evidence of efficacy of curcumin C3 complex. Data suggest limited bioavailability	[424]
	Curcumin	36 patients with dementia, presumed AD	1 g curcumin + 120 mg ginkgo leaf extract, 4 g curcumin + ginkgo leaf extract or placebo daily for 6 months	Change in isoprostane levels in plasma and change in beta-amyloid levels in serum	Serum beta-amyloid rose on curcumin. Fewer adverse events reported	[425]
	Quercetin	48 patients with MCI or early AD	1000 mg quercetin or 100 mg dasatinib or placebo daily for 2 days	Serious adverse events and adverse events, and change in cellular senescence blood markers	Ongoing	[426]
	Quercetin	Recruiting patients with early AD	Quercetin + dasatinib for 2 days on, 14 days off for 12 weeks (6 cycles)	Brain penetrance of dasatinib and quercetin	Ongoing	[427]
	EGCG	21 patients with early AD	200 mg, 400 mg, 600 mg and 800 mg EGCG tri-monthly or placebo for 18 months	ADAS-Cog	Ongoing	[428]
	EGCG	200 patients with AD carrying ApoE4 allele	260–520 mg EGCG + personalized intervention or placebo + non personalized intervention or 260–520 mg EGCG + non personalized intervention or placebo to personalized intervention, daily for 15 months	Evaluate the efficacy of multimodal intervention (dietary, physical and cognition) combined with EGCG in slowing down cognitive decline	Ongoing	[429]
	Genistein	27 patients with mild AD	60 mg genistein or placebo daily for 360 days	Changes in amyloid beta concentration of CSF	Ongoing	[430]
	Genistein + Daidzein	72 patients with AD	100 mg of soy isoflavones or placebo daily for 6 months	Cognitive outcomes: language execution function, verbal memory and recall, attention, visual memory and planning	Six months of 100 mg/day isoflavones did not benefit cognition in older men and women with AD	[431]
	Alpha-Lipoic Acid + Omega-3 Fatty Acids	39 patients with mild AD	600 mg alpha-lipoic acid + 3 g fish oil, 3 g fish oil alone or placebo daily for 12 months	Peripheral F2-isoprostane levels (oxidative stress measure)	Combination of alpha lipoic acid with omega-3 fatty acids slowed cognitive and functional decline	[432]
Minerals	Copper	68 patients with mild to moderate AD	8 mg copper or placebo daily for 1 year	Change in cognitive function, measured by ADAS-Cog	Results not yet published	[433]
	Selenium + Vitamin E	7540 participants with dementia	200 μg Selenium + 400 IU Vitamin E, 200 μg selenium + placebo or 400 IU vitamin E + placebo or placebo + placebo daily for 7–12 years	Incidence of dementia (including AD)	Neither supplement prevented dementia	[434]
